# Lumbar intervertebral disc degeneration and related factors in Korean firefighters

**DOI:** 10.1136/bmjopen-2016-011587

**Published:** 2016-06-28

**Authors:** Tae-Won Jang, Yeon-Soon Ahn, Junsu Byun, Jong-In Lee, Kun-Hyung Kim, Youngki Kim, Han-Soo Song, Chul-Gab Lee, Young-Jun Kwon, Jin-Ha Yoon, Kyoungsook Jeong

**Affiliations:** 1 Department of Occupational and Environmental Medicine, Korea University Ansan Hospital, Ansan, Korea; 2 Department of Occupational and Environmental Medicine, Dongguk University Ilsan Hospital, Goyang, Korea; 3 Department of Occupational and Environmental Medicine, College of Medicine, Catholic University of Korea, Seoul, Korea; 4 Department of Occupational and Environmental Medicine, Busan Paik Hospital, Inje University, Busan, Korea; 5 Department of Occupational and Environmental Medicine, Busan National University Yangsan Hospital, Yangsan, Korea; 6 Department of Occupational and Environmental Medicine, School of Medicine, Chosun University, Gwangju, Korea; 7 Department of Occupational and Environmental Medicine, Hallym University Sacred Heart Hospital, Anyang, Korea; 8 Department of Preventive Medicine and Public Health, Yonsei University College of Medicine, Seoul, Korea

**Keywords:** Degeneration, Firefighter, Intervertebral disc, Occupation

## Abstract

**Objectives:**

The job of firefighting can cause lumbar burden and low back pain. This study aimed to identify the association between age and lumbar intervertebral disc degeneration and whether the association differs between field and administrative (non-field) firefighters.

**Methods:**

Subjects were selected using a stratified random sampling method. Firefighters were stratified by geographic area, gender, age and type of job. First, 25 fire stations were randomly sampled considering regional distribution. Then firefighters were stratified by gender, age and their job and randomly selected among the strata. A questionnaire survey and MRI scans were performed, and then four radiologists used Pfirrmann classification methods to determine the grade of lumbar intervertebral disc degeneration.

**Results:**

Pfirrmann grade increased with lumbar intervertebral disc level. Analysis of covariance showed that age was significantly associated with lumbar intervertebral disc degeneration (p<0.05). The value of β (parameter estimate) was positive at all lumbar intervertebral disc levels and was higher in the field group than in the administrative group at each level. In logistic regression analysis, type of job was statistically significant only with regard to the L4–5 intervertebral disc (OR 3.498, 95% CI 1.241 to 9.860).

**Conclusions:**

Lumbar intervertebral disc degeneration is associated with age, and field work such as firefighting, emergency and rescue may accelerate degeneration in the L4–5 intervertebral disc. The effects of field work on lumbar intervertebral disc degeneration were not clear in discs other than at the level L4–5.

**Strengths and limitations of this study**

- The study was well designed with a stratified random sampling method.
- We measured intervertebral disc degeneration using MRI, which is the most accurate method for clinical assessment of this condition.
- Genetics and some medical conditions, such as diabetes and dyslipidaemia, have been found to be associated with lumbar intervertebral disc degeneration, but were not investigated in this study.

## Introduction

The intervertebral disc is a basic structure that lies between two vertebrae. Intervertebral discs buffer mechanical stress on the spine and enable spinal flexion, extension, bending and rotation.^1^ Lumbar intervertebral discs degenerate with age.^1^ ^2^ Degeneration is a major cause of low back pain,^3^ ^4^ may lead to sensory disturbance and weakness in the legs, disability and poor quality of life,^5^ ^6^ and imposes high economic burden.^7^

Older age is associated with an increased prevalence of lumbar intervertebral disc degeneration.^8^ Genetic factors^9–12^ and modifiable factors, such as obesity,^13^ ^14^ smoking,^15^ diabetes,^16^ atherosclerosis,^16^ ^17^ dyslipidaemia,^13^ bone mineral density,^18^ occupation^13^ ^19^ and sport,^13^ ^19^ ^20^ are also associated with lumbar intervertebral disc degeneration.

Sources of occupation-related physical stress include handling of heavy materials, awkward posture, heavy physical work and whole-body vibration.^21^ Many researchers consider work-related factors to be associated with risk of lumbar intervertebral disc degeneration. However, some studies suggest that genetic factors are more strongly associated, and work-related factors play a relatively minor role.^2^ Furthermore, others report no association between work-related factors and lumbar intervertebral disc degeneration.^22^

Although work-related factors, such as handling of heavy material and awkward posture, may be associated with degenerative diseases of the lumbar spine, few studies have investigated the association between work-related factors and lumbar intervertebral disc degeneration. The present study aimed to identify the association between age and lumbar intervertebral disc degeneration and whether the association differs according to different work-related lumbar burden between field and administrative (non-field) firefighters.

## Methods

### Subjects

The design of the study was cross-sectional. We selected subjects using a stratified random sampling method in order to improve the representativeness of the subjects. Initially, 25 fire stations were randomly sampled considering regional distribution. We then stratified firefighters by gender, age (20–29, 30–39, 40–49 and 50–59) and type of job (firefighting, emergency, rescue and administrative). The statements of PROC SURVEY, PROC SORT and PROC RANK in SAS Windows V.9.2 were used to generate random numbers and determine the priority of the firefighters in each stratified group. Exclusion criteria were retirement during the study period, failure to complete the questionnaire survey, and contraindication for radiological examination (such as pregnancy and other medical diseases). If the subject selected as first priority was unable to participate, the subject selected as second priority was chosen as a participant. A total of 442 firefighters were selected as participants. After exclusion of 50 subjects with insufficient information, 392 were evaluated. Sample size was calculated with 0.05 of α error and 0.8 of power using G*Power Windows V.3.1. The study was carried out from May 2014 to March 2015. It was approved by the Institutional Review Board of Dongguk University Ilsan Hospital (approval ID 2014-82). Informed consent was obtained from all study subjects.

### Methods

The questionnaire survey, which included general and work-related characteristics, was administered to all participants. General characteristics included age, gender, height, weight, marital status, education, income, alcohol drinking, smoking, exercise and hours spent performing housework. Work-related characteristics included job duration, work schedule and type of job. For alcohol drinking, more than one bottle of Soju (72 g alcohol) consumed in a week was considered moderate or heavy drinking. Rotating two-shift or three-shift work was considered to be shift work.

MRI is the most accurate method for clinical assessment of intervertebral disc pathology. The signal intensity of the intervertebral disc in T2-weighted MRI reflects the degree of degeneration.^23^ MRI scans were conducted on all study subjects at the five hospitals, using an MRI protocol that was defined before the scans were performed to maintain consistency across all subjects. Four radiologists read MRI scans and determined the grade of degeneration for each level of lumbar intervertebral disc according to Pfirrmann classification, which assesses degeneration by grading (on a scale ranging from 1 to 5) the structure of the disc, distinction of nucleus from annulus, signal intensity, and height of intervertebral disc visualised on MRI.^24^ Each MRI scan was assigned to two radiologists. In cases of inconsistency, the higher Pfirrmann grade was selected.

### Statistical analysis

The field group (n=273) included firefighters with a firefighting, emergency or rescue job, and the administrative group (n=119) included those who carried out administration. We conducted analysis of covariance to identify the association between Pfirrmann grade and age. Analysis of covariance was performed for all subjects, the field group and the administrative group. Pfirrmann grade was the dependent variable, and age, job duration, gender, body mass index (BMI), socioeconomic status and work-related characteristics were the independent variables. Logistic regression analysis was performed with Pfirrmann grade as the dependent variable. Pfirrmann grade was categorised as low (grade 1–2 in lumbar intervertebral disc level L1–2, L2–3 and L3–4, and grade 1–3 in L4–5 and L5–S1) and high (grade 3–5 in L1–2, L2–3 and L3–4, and grade 4–5 in level L4–5 and L5–S1) according to the distribution of Pfirrmann grade at each level. Statistical analysis was conducted using SAS Windows V.9.2. Statistical significance was set at p<0.05.

## Results

Table 1 shows the general and work-related characteristics of the subjects. Age distributions were similar between the field and administrative groups. The field group had a greater proportion of men (81.0%) than the administrative group (68.9%) (p<0.05). Prevalence of obesity (BMI ≥25 kg/m^2^) tended to be higher in the field group (35.5%) than in the administrative group (25.2%), but the difference was not statistically significant. Marital status, monthly income, alcohol drinking, smoking and exercise were similar between the two groups. The field group had a greater proportion of individuals with a high level of education than did the administrative group (p<0.05). Job duration was similar between the two groups, although the field group had a greater proportion of shift workers (p<0.05).

**Table 1:** General and work-related characteristics of field and administrative groups

Variable	Field (n=273)	Administrative (n=119)
Age (years)		
<30	65 (23.8)	28 (23.5)
30–39	88 (32.2)	42 (35.3)
40–49	70 (25.7)	27 (22.7)
≥50	50 (18.3)	22 (18.5)
Gender*		
Male	221 (81.0)	82 (68.9)
Female	52 (19.0)	37 (31.1)
Body mass index (kg/m^2^)*		
<25	176 (64.5)	89 (74.8)
≥25	97 (35.5)	30 (25.2)
Marital status		
Unmarried	81 (29.7)	29 (24.4)
Married	190 (69.6)	89 (74.8)
Divorced or widowed	2 (0.7)	1 (0.8)
Education*		
High school or less	64 (23.4)	21 (17.7)
College	107 (39.2)	35 (29.4)
University	102 (37.4)	63 (52.9)
Monthly income (KRW)		
<3 000 000	119 (44.3)	56 (47.5)
3 000 000–4 999 999	123 (45.7)	47 (39.8)
≥5 000 000	27 (10.0)	15 (12.7)
Alcohol drinking		
None or social	99 (36.4)	38 (31.9)
Moderate or heavy†	173 (63.6)	81 (68.1)
Smoking		
Never smoker	127 (46.5)	61 (51.3)
Ex-smoker	76 (27.9)	32 (26.9)
Current smoker	70 (25.6)	26 (21.8)
Exercise (frequency/week)‡		
0	21 (8.2)	12 (11.2)
1	58 (22.8)	30 (28.0)
≥2	176 (69.0)	65 (60.8)
Housework (hours/day)		
Negligible	80 (29.3)	45 (38.1)
<1	119 (43.6)	47 (39.8)
1–2	52 (19.1)	20 (17.0)
≥2	22 (8.0)	6 (5.1)
Job duration (years)		
<10	139 (50.9)	52 (43.7)
10–19	69 (25.3)	37 (31.1)
≥20	65 (23.8)	30 (25.2)
Work schedule*		
Day work	15 (5.5)	110 (93.2)
Shift work§	257 (94.5)	8 (6.8)

Data are shown as number (%).

*p<0.05.

†Drinking more than 1 bottle of Soju (72 g alcohol) per week.

‡Moderate or vigorous intensity of leisure-time exercise.

§Rotating two-shift or three-shift work.

Table 2 shows the Pfirrmann grade according to the level of lumbar intervertebral disc. Pfirrmann grade increased with lumbar intervertebral disc level. The distribution of Pfirrmann grade was not significantly different between the field and administrative groups at any lumbar intervertebral disc level (p>0.05).

**Table 2:** Pfirrmann grade distribution between field and administrative groups

Disc level and Pfirrmann grade	All (n=392)	Field (n=273)	Administrative (n=119)
L1–2			
Grade 1	203 (51.8)	140 (51.3)	63 (52.9)
Grade 2	89 (22.7)	59 (21.6)	30 (25.2)
Grade 3	87 (22.2)	66 (24.2)	21 (17.7)
Grade 4	13 (3.3)	8 (2.9)	5 (4.2)
L2–3			
Grade 1	179 (45.7)	119 (43.6)	60 (50.4)
Grade 2	95 (24.2)	68 (24.9)	27 (22.7)
Grade 3	80 (20.4)	60 (22.0)	20 (16.8)
Grade 4	36 (9.2)	25 (9.1)	11 (9.3)
Grade 5	2 (0.5)	1 (0.4)	1 (0.8)
L3–4			
Grade 1	146 (37.2)	97 (35.5)	49 (41.2)
Grade 2	102 (26.0)	74 (27.1)	28 (23.5)
Grade 3	94 (23.5)	66 (24.2)	28 (23.5)
Grade 4	50 (12.8)	36 (13.2)	14 (11.8)
L4–5			
Grade 1	70 (17.8)	48 (17.6)	22 (18.5)
Grade 2	63 (16.1)	47 (17.2)	16 (13.4)
Grade 3	110 (28.1)	74 (27.1)	36 (30.3)
Grade 4	143 (36.5)	100 (36.6)	43 (36.1)
Grade 5	6 (1.5)	4 (1.5)	2 (1.7)
L5–S1			
Grade 1	88 (22.5)	58 (21.2)	30 (25.2)
Grade 2	63 (16.1)	42 (15.4)	21 (17.7)
Grade 3	87 (22.2)	63 (23.1)	24 (20.2)
Grade 4	144 (36.7)	101 (37.0)	43 (36.1)
Grade 5	10 (2.5)	9 (3.3)	1 (0.8)

Data are shown as number (%). L and S refer to lumbar and sacral vertebrae, respectively.

Table 3 shows the results of the analyses of covariance. Age was significantly associated with Pfirrmann grade except for L1–2, L3–4 and L5–S1 intervertebral disc levels in the administrative group (p<0.05). The value of β (parameter estimate) was positive at all lumbar intervertebral disc levels and was higher in the field group than in the administrative group at each level.

**Table 3:** Association between Pfirrmann grade and age in analysis of covariance

	All (n=392)		Field (n=273)		Administrative (n=119)	
Disc level	β	p Value	β	p Value	β	p Value
L1–2	0.02020	0.0001	0.02445	0.0002	0.01482	0.1275
L2–3	0.03387	<0.0001	0.03655	<0.0001	0.02645	0.0154
L3–4	0.03926	<0.0001	0.04693	<0.0001	0.01915	0.0991
L4–5	0.04773	<0.0001	0.05504	<0.0001	0.03398	0.0053
L5–S1	0.03822	<0.0001	0.04532	<0.0001	0.02584	0.0547

Data are β and p value of age in analysis of covariance (adjusted for gender, body mass index, socioeconomic status and work-related variables). L and S refer to lumbar and sacral vertebrae, respectively.

Table 4 shows the results of the logistic regression analyses. OR for age was <1.0 at each lumbar disc level (p<0.05), whereas type of job was significant only at L4–5 (p<0.05). OR for field work at this level was 3.498 (95% CI 1.241 to 9.860).

**Table 4:** Association between Pfirrmann grade and related factors

Disc level	Variable	OR* (95% CI)
L1–2	Age	0.942 (0.917 to 0.968)
	Type of job	
	Administrative	1.000
	Field	1.194 (0.442 to 3.228)
L2–3	Age	0.917 (0.892 to 0.943)
	Type of job	
	Administrative	1.000
	Field	1.333 (0.500 to 3.551)
L3–4	Age	0.938 (0.905 to 0.973)
	Type of job	
	Administrative	1.000
	Field	1.734 (0.483 to 6.226)
L4–5	Age	0.938 (0.914 to 0.962)
	Type of job	
	Administrative	1.000
	Field	3.498 (1.241 to 9.860)
L5–S1	Age	0.951 (0.927 to 0.974)
	Type of job	
	Administrative	1.000
	Field	1.181 (0.470 to 2.969)

L and S refer to lumbar and sacral vertebrae, respectively.

*Adjusted for age, gender, body mass index, socioeconomic status and work-related variables.

## Discussion

This study showed that lumbar intervertebral disc degeneration increased with age and that field work such as firefighting, emergency and rescue may accelerate the effect of age on degeneration at the level L4–5.

Age is known to be associated with lumbar intervertebral disc degeneration. Powell *et al*^8^ reported that 30% of 20–29-year-old subjects and 90% of 70–79-year-old subjects in their study had lumbar intervertebral disc degeneration. Zheng and Chen^4^ showed that lumbar intervertebral disc degeneration and low back pain increase with age. Boos *et al*^25^ reported a steady increase in intervertebral disc degeneration scores with age in an histological study of deceased subjects (age range 0–88 years). Signal intensity, narrowing of disc height, disc bulging or herniation, osteophytes and fatty degeneration are also positively associated with age.^26^ In the present study, lumbar disc degeneration measured by Pfirrmann grade was associated with age in all subjects and in the field group, whereas the association was not significant in L1–2, L3–4 and L5–S1 intervertebral discs in the administrative group. Therefore, an increased tendency to lumbar intervertebral disc degeneration with age was clear in the firefighters engaged in field work. The results in the administrative group may be explained by the relatively small number in that group.

Firefighting is a hazardous occupation with high physical demands and levels of psychological stress.^27^ It has been reported to show very high physical activity levels compared with sedentary jobs.^28^ A study by Ainsworth *et al*^29^ showed that energy expended in firefighting is 6.8–9.0 metabolic equivalents, which classifies it as a very physically demanding occupation.^30^ Fire suppression tasks impose significant musculoskeletal demand because of the awkward postures required, such as severe trunk flexion.^31^ According to evaluation with ergonomic tools such as the National Institute of Occupational Safety Health (NIOSH) Lifting Equation, Rapid Entire Body Assessment (REBA) and Rapid Upper Limb Assessment (RULA), emergency medical services require frequent bending or twisting of the lower back.^32^ Broniecki *et al*^33^ reported that emergency medical services require strenuous physical work, including frequent lifting, pushing, pulling and carrying of patients, and this can increase lumbar burden. The lumbar burden resulting from firefighting may contribute to the high prevalence of low back pain and low back injury in firefighters.^34^ In the present study, the value of β in the analysis of covariance was higher in the field group than in the administrative group at every lumbar intervertebral disc level, indicating that the degree of lumbar intervertebral disc degeneration was higher in the field group than in the administrative group. In the logistic regression analysis, type of job was statistically significant only at the lumbar intervertebral disc level L4–5. This suggests that lumbar intervertebral disc degeneration associated with age may be accelerated by work-related lumbar burden at this level.

Intervertebral disc degeneration usually begins during the second decade of human life and occurs more rapidly than degeneration of other spinal structures.^35^ Histological changes involved in disc degeneration include mucoid degeneration, granular changes, tears and cleft formation,^25^ and macroscopic changes include alterations in nucleus pulposus, annulus fibrosus, endplates and vertebral body.^36^ Adams and Roughley^37^ indicate that the most important cause of intervertebral disc degeneration may be the processes that weaken a disc or impair its healing response, such as genetic predisposition, aging, malnutrition and mechanical loading. In the present study, lumbar intervertebral disc degeneration was greater in field firefighters than in administrative firefighters, possibly because the former experienced more mechanical loading of the lower back which may have accelerated degeneration of the lumbar intervertebral discs.

Intervertebral disc degeneration may be associated with low back pain and disc herniation.^38^ Low back pain and disorders (such as herniated lumbar intervertebral discs) are prevalent in workers who undergo mechanical loading on the lumbar spine. Epidemiological studies have shown a higher prevalence of low back disorders in workers who have high mechanical loads on the lumbar spine from handling heavy materials, awkward postures, heavy physical work and whole-body vibrations.^21^ ^39^ However, the relationship between work-related factors and lumbar intervertebral disc degeneration is not as clear as the association of the latter with age or genetics. Many studies have investigated the relationship between work-related factors and low back pain or disorders, but few have analysed the morphology or biomechanics of intervertebral disc pathology or used MRI to investigate intervertebral disc degeneration. Because lumbar intervertebral disc degeneration is related to low back disorders,^39^ many researchers assume work-related factors are related to lumbar intervertebral disc degeneration. Although many studies report a significant association between intervertebral disc degeneration and work-related factors,^13^ ^40^ others do not.^41^ ^42^

Recent studies report that genetic factors play a major role in, and are clearly associated with, lumbar intervertebral disc degeneration.^9–12^ However, genetics and age are not modifiable factors. Identification of modifiable factors associated with lumbar intervertebral disc degeneration, including obesity,^13^ ^14^ smoking,^15^ medical conditions such as diabetes and dyslipidaemia,^13^ ^16^ bone mineral density,^18^ occupation^13^ ^19^ and sport,^13^ ^19^ ^20^ is important in preventing intervertebral disc disorders. In the present study, lumbar intervertebral disc degeneration was greater in field firefighters than administrative firefighters, suggesting that mechanical loading accelerates lumbar intervertebral disc degeneration and that reducing the load may help prevent related disorders.

There are some limitations of the present study. First, genetics and some medical conditions, such as diabetes and dyslipidaemia, have been found to be associated with intervertebral disc degeneration, but were not investigated. Therefore, we were unable to identify the effect of these factors on intervertebral disc degeneration or adjust for them in statistical analyses. Second, work-related factors that may be associated with lumbar intervertebral disc degeneration, such as duration of firefighting and driving of fire engines or ambulances, was not investigated.

The present study also has several strengths. First, it was well designed with a stratified random sampling method. Second, we measured intervertebral disc degeneration using MRI, which is the most accurate method for clinical assessment of this condition.

Lumbar intervertebral disc degeneration is associated with age, and work-related mechanical lumbar loading may accelerate it at the level L4–5. Firefighters who perform field activities such as firefighting, emergency and rescue that place a high physical demand on the lower back may have an increased risk of lumbar intervertebral disc degeneration at the level L4–5, which may contribute to low back pain and intervertebral disc herniation. The effects of field work on degeneration in lumbar intervertebral discs other than at the level L4–5 were not clear. Further studies are needed to investigate the effect of mechanical loading on lumbar intervertebral disc degeneration in workers engaged in other types of occupation.
